# Reward feedback stimuli elicit high-beta EEG oscillations in human dorsolateral prefrontal cortex

**DOI:** 10.1038/srep13021

**Published:** 2015-08-17

**Authors:** Azadeh Haji Hosseini, Clay B. Holroyd

**Affiliations:** 1 PhD Student, Department of Psychology, University of Victoria, V8W 2Y2, Canada; 2 Professor and Canada Research Chair, Department of Psychology, University of Victoria, V8W 2Y2, Canada

## Abstract

Reward-related feedback stimuli have been observed to elicit a burst of power in the beta frequency range over frontal areas of the human scalp. Recent discussions have suggested possible neural sources for this activity but there is a paucity of empirical evidence on the question. Here we recorded EEG from participants while they navigated a virtual T-maze to find monetary rewards. Consistent with previous studies, we found that the reward feedback stimuli elicited an increase in beta power (20–30 Hz) over a right-frontal area of the scalp. Source analysis indicated that this signal was produced in the right dorsolateral prefrontal cortex (DLPFC). These findings align with previous observations of reward-related beta oscillations in the DLPFC in non-human primates. We speculate that increased power in the beta frequency range following reward receipt reflects the activation of task-related neural assemblies that encode the stimulus-response mapping in working memory.

Neural oscillations in the ongoing electroencephalogram (EEG) are believed to reflect the synchronous activity of distributed neuronal cell assemblies that encode distinct neurocognitive functions^1^,^2^. In particular, several studies have reported that reward-related feedback stimuli elicit increased power in the high-beta frequency range (20–35 Hz) in the human EEG and magnetoencephalogram over frontal areas of the scalp^3^,^4^,^5^ (hereafter called “beta”). Enhanced frontal beta power is also elicited by unexpected reward feedback stimuli compared to expected reward feedback stimuli^6^ and by the first positive feedback stimulus compared to subsequent positive feedback in the Wisconsin Card Sorting Test^7^. Consistent with these findings, it has recently been proposed that reward-related beta serves to couple attentional and emotional systems associated with novelty and reward processing^8^, and that beta oscillations play a role in synchronizing neural activity to promote learning from positive feedback^9^,^10^. However, the specific role of these oscillations in reward processing is still poorly understood.

Insight into the functionality of these oscillations could be derived from identifying their neural origin^8^,^10^. Previously, we found that reward-related feedback stimuli elicit an increase in beta power over right-frontal areas of the human scalp, and speculated that this signal could be produced by right dorsolateral prefrontal cortex (DLPFC)^11^. Consistent with this possibility, studies in non-human primates have revealed beta oscillations in the principal sulcus, a homologue of human DLPFC that is associated with rule implementation and category learning^12^,^13^,^14^. However, whether human DLPFC produces beta oscillations is unknown.

Here we recorded the EEG from participants engaged in a reinforcement learning task in which they navigated a virtual T-maze to find monetary rewards, and applied a source localization technique to investigate possible generators of reward-related beta oscillations. We predicted that rewards compared to errors would elicit higher beta power over frontal areas of the scalp and that this contrast would be localized to the DLPFC.

## Results

EEG was recorded from 26 undergraduate students while they engaged in a virtual T-maze task with reinforcement. On each trial they were presented with a visual cue and then entered either a left or right alley in the maze by pressing a corresponding button. A feedback stimulus indicating monetary reward or error (no-reward) was presented at the end of the trial. The feedback stimuli were probabilistically associated with different cue-response combinations such that some conditions were relatively easy to learn, yielding high probability rewards and low probability errors (easy condition), and other conditions were relatively difficult to learn, yielding high probability errors and low probability rewards (hard condition). Subjects were instructed to utilize the feedback to maximize their earnings. See methods for a complete description.

## Behavioral analysis

Participants selected the rewarding arm of the maze on 68.2 ± 0.1% of the trials overall, on 74.3 ± 0.1% of the trials in the easy condition (high probability of reward, low probability of error), and on 62.0% ± 0.1% of the trials in the hard condition (low probability of reward, high probability of error). The number of visits to rewarding arms was significantly higher for the easy condition relative to the hard condition (*t*(25) = 6.20, *p* < 0.001). Average reaction time was 275 ± 85 ms across conditions, with no statistically significant difference between the conditions. Note that the participants were not permitted to respond until the appearance of the response cue, 1000 ms following the onset of the stimulus cue, which likely accounts for the uniformity of the reaction times across the stimulus conditions.

## Time-frequency analysis

**Figure 1:**
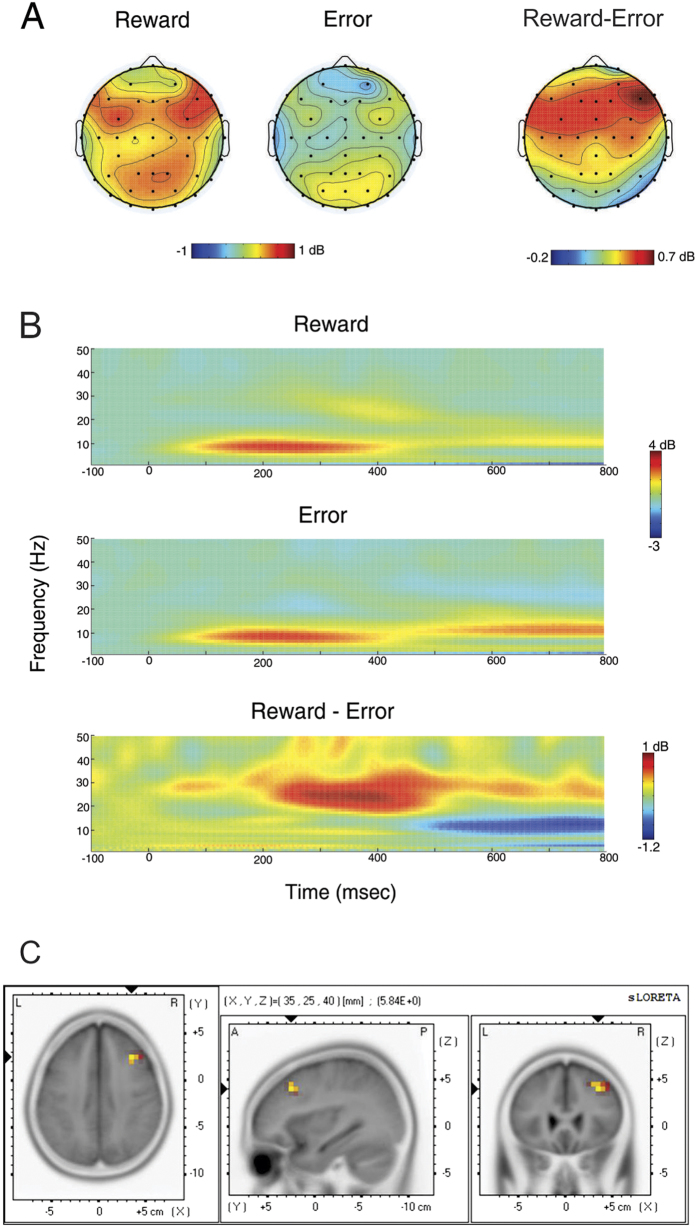
Time-frequency and sLORETA source localization results. (**A**) scalp distribution of beta (20–30 Hz) power in reward (left), error (middle), and reward-error conditions. (**B**) time-frequency maps of power for the reward (top), error (middle), and reward-error (bottom) conditions at channel F6. (**C**) Right dorsolateral prefrontal cortex was revealed as the source of beta (25 Hz) contrast, reward vs. error, averaged over 250–450 ms post-feedback.

A 9 × 2 × 2 ANOVA on beta power with channel (F5, FZ, F6, C5, CZ, C6, P5, PZ, P6), valence (reward, error), and probability (high, low) as factors revealed a significant effect of valence (*F*(1,25) = 18.25, *p* < 0.001), a significant interaction of channel and valence (*F*(8,200) = 2.91, *p* = .023), and no other main effects or interactions. Figure 1A illustrates the scalp distribution of beta power for the reward and error conditions and for the difference between the two conditions; note that the difference was distributed over right-frontal areas of the scalp, reaching a maximum value at channel F6. A 2 × 2 ANOVA on beta power associated with channel F6 confirmed the effect of valence (*F*(1,25) = 32.51, *p* < .001) and no effect of probability or interactions with probability and valence at that channel. Figure 1B presents time-frequency maps of power for reward and error conditions, and their difference, associated with channel F6.

## Source localization

Source analysis was applied to the observed valence effect on beta (see methods). Figure 1C illustrates the location of the maximum t-value for the valence-related effect of beta power (*t* = 5.84, *p* = 0.001; X = 35, Y = 25, Z = 40, MNI coordinates), corresponding to Brodmann area 9 in the middle frontal gyrus of right DLPFC.

## Discussion

Frontal beta oscillations are elicited by reward-related feedback stimuli, but not by non-reward or error-related feedback stimuli, over frontal areas of the scalp^4^,^5^,^6^. Current proposals suggest that reward-related beta is generated within dorsal anterior cingulate cortex^8^,^10^ or ventromedial prefrontal cortex^8^. However, recent investigations have indicated a focus over right prefrontal areas^6^,^7^,^11^ , suggesting that reward beta might originate from right prefrontal cortex. Here we verified this supposition with what is to our knowledge the first empirical investigation on source-localization of reward-related beta oscillations in humans. Our results replicated the previously observed sensitivity of frontal beta to valence and further implicated right DLPFC as the neural generator, as predicted.

Current theories of reward-related beta oscillations have variously suggested that the signal might reflect a neural mechanism for learning from feedback^10^, for synchronizing neural activity to promote learning from positive feedback^9^, and for coupling systems involved in memory, attention, and motivation^8^. Further, an influential theory of working memory proposes that maintenance of individual items in working memory is mediated by interacting beta and theta oscillations^15^. This theory has been supported by observations that beta-gamma oscillations in the human frontal cortex and hippocampus scale with working memory load^16^ and couple with oscillations in theta range as predicted by the theory^17^. In view of the well-known role of human DLPFC in maintaining task-related information in working memory^18^,^19^,^20^, our results suggest that beta oscillations mediate a link between DLPFC processes related to reward learning and working memory.

In line with these observations, neurons on the banks of the monkey principal sulcus, a homologue of human DLPFC, are active in a rule-specific manner depending on task requirements^14^, and code for the currently relevant task rule by synchronizing in the beta frequency range^13^. Synchrony in the beta frequency range between monkey striatal and PFC neurons also increases during category learning^12^, suggesting that beta oscillations may facilitate communication between the PFC and striatum during such learning.

In the context of this literature, we speculate that increased power in the beta frequency range following reward receipt reflects enhanced activation of task-related neural assemblies that encode the stimulus-response mapping for that trial^21^. On this view, the synchronous activity at the beta frequency range of neurons in DLPFC and the striatum would facilitate the transfer of rewarded action sequences to other brain areas^12^,^22^,^23^. Once learned, these sequences could be executed automatically, reducing the need for communication of task demands placed on the DLPFC (e.g. Cunillera *et al.*, 2012^7^)^24^,^25^, a process that would complement other proposed mechanisms for integrating working memory with reinforcement learning^25^,^26^. This hypothesis could be tested by disrupting or enhancing reward-related beta oscillations in human DLPFC using non-invasive stimulation techniques such as transcranial magnetic stimulation or transcranial direct current stimulation^27^,^28^.

## Method

### Participants

Twenty-six undergraduate students (7 men, 20.3 ± 3.8 years old) at the University of Victoria participated in the experiment. Subjects acquired extra course credits for participation and were also paid a monetary bonus that depended on task performance. The study was conducted in accordance with the ethical standards prescribed in the Declaration of Helsinki and was approved by the human subjects review board at the University of Victoria. Informed written consent was obtained from all participants prior to the experiment.

### Task

**Figure 2:**
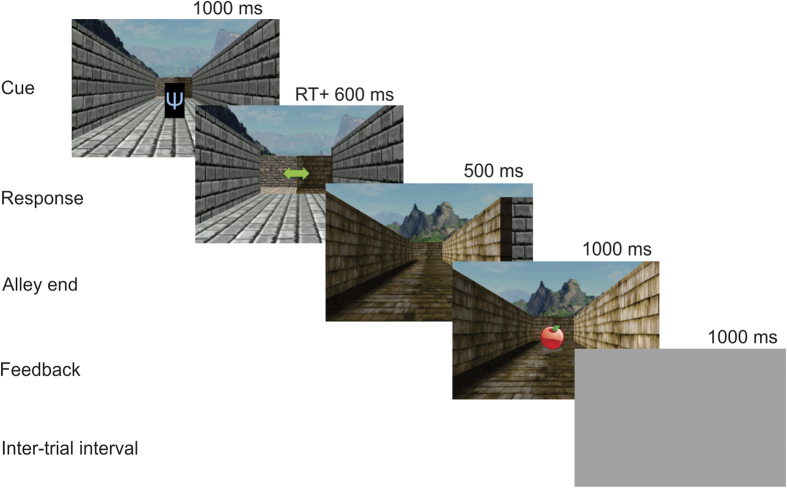
Virtual T-maze task. Participants were instructed to navigate a virtual maze by choosing a left or right response according to visual cues presented at the start of each trial. The cue was presented over an image of the stem alley for 1000 ms (“Cue”). Then, an image of a double arrow appeared on the screen and remained on the screen until 600 ms after a response was selected (“Response”). A view of the selected alley was then presented for 500 ms (“Alley end”), followed by a closer view of the end of the alley with an image of the feedback stimulus (apple or orange) overlaid at central fixation (“Feedback”), indicating that participants earned either 5 cents (reward) or 0 cents (error). A blank screen was presented for 1000 ms between trials (“Inter-trial interval”). See methods for the probability mappings between cues, responses and feedback stimuli. We used Gamestudio and Microsoft Office to create and edit the stimuli.

Participants performed a version of a virtual T-Maze task used previously to investigate reward-related electrophysiological activity^29^, modified according to probabilistic stimulus-reward contingencies derived from Holroyd, Krigolson, Baker, Lee & Gibson (2009)^30^. Note that a previous experiment in which participant responses were rewarded at random on 50% of the trials failed to produce reward-related beta oscillation. We therefore modified the task such that the feedback depended probabilistically on prior stimuli and responses, in the expectation that beta power would be enhanced by an increase in perceived control over the trial outcomes. Subjects were instructed to navigate the virtual T-maze according to visual cues presented at the start of each trial. Figure 2 illustrates the event timing for an example trial in the task. At the beginning of each trial, a visual cue belonging to one of several categories (described below) was presented over an image of the stem alley. To convey a sense of movement, 1000 ms later the stimuli were replaced by an image that showed a closer view of the end of the alley superimposed by a double-arrow (Fig. 2). Upon seeing the arrow participants were instructed to select the right or left alley by pressing the corresponding arrow key on the keyboard. To limit the overall duration of the experiment (as opposed to pressing the participants for speed), responses that exceeded 1s were penalized with a 25 cents loss. Participants were not informed about the specific deadline but were instructed that slow responses would result in the loss. 600 ms after the response, an image of the chosen alley was presented for 500 ms, followed by a closer view of the end of the alley, overlaid with an image of the feedback stimulus (5.5^o^ of visual angle) at central fixation, presented for 1000 ms. Participants were told that that if they found an apple (orange) then they gained 5 cents on that trial and if they found an orange (apple) then they gained 0 cents on that trial; the assignation of reward values to feedback stimuli was counterbalanced across participants.

The task consisted of three blocks of trials, each characterized by a different set of four possible shapes for the initial cue. These three stimulus sets consisted of four geometrical shapes (square, triangle, circle, and trapezoid), four black squares depicting letters from the Greek alphabet (β, π, ψ, and Σ), and four cartoon sky-related shapes (sun, moon, star, and cloud). On each trial the cue was randomly chosen without replacement from the set of four. To prevent against the development of irrelevant stimulus associations, the stimulus colors differed across stimuli both within and across blocks. Within each block, each of the four shapes corresponded to a specific alley-probability combination, determined at random: 70% reward probability for right alley choices, 70% reward probability for left alley choices, 30% reward probability for right alley choices, and 30% reward probability for left alley choices. The opposite alley in all four stimulus conditions was never rewarded (0% probability of reward). Thus, for each cue only one alley was rewarded and the probability of reward on that alley was either low or high, resulting in a two-by-two task design with levels for valence (reward, error) and probability (low, high).

We wrote our experiment in Matlab, using the Psychophysics Toolbox extensions^31^.

### Data acquisition

The EEG was recorded from 51 electrode locations using BrainVision Recorder software. Electrodes were arranged according to the standard 10–20 layout^32^ and were referenced online to the average voltage across the channels. Vertical and horizontal ocular movements were recorded by an electrode placed under the right eye (re-referenced offline to FP2), and two on the outer canthi of the right and left eyes (re-referenced offline to each other) respectively. Electrode impedances were kept under 10 kΩ. Data were sampled at 500 Hz and high pass filtered online at 0.017 Hz.

### Data analysis

Data pre-processing was performed in BrainVision Analyzer 2. A band-pass filter (0.1–100 Hz) was applied to the EEG data and epochs of EEG activity were selected from 1 s before to 1 s after the onset of feedback stimuli. Data were subsequently re-referenced to the average value recorded at the mastoids. Ocular correction was performed using the Gratton, Coles, and Donchin (1983)^33^ algorithm as implemented in the Analyzer software. Feedback segments were baseline-corrected by subtracting the average voltage values during the 100 ms prior to the feedback stimulus from the value of each sample in the epoch, for each channel, subject, and electrode. EEG artifacts were identified and rejected according to the following criteria: Any abrupt change of voltage greater than 35 μV across consecutive samples, any difference between the negative and positive peaks in a 200 ms interval that exceeded 150 μV, and any activity that was consistently smaller than 0.5 μV in a 100 ms interval were considered artifacts and the corresponding segment was rejected for all channels. On average, 7% of data were discarded. Data were then exported to MATLAB for the ERP and time-frequency analyses. Topographical scalp maps were plotted with EEGLAB^34^.

To extract time-frequency information, for each subject, trial and channel, a two-second epoch centered on the time of feedback presentation was convoluted with a seven-cycle complex Morlet wavelet. The wavelet was linearly scaled based on the frequency range of 1–50 Hz and the power for each frequency band was evaluated relative to the 100 ms baseline before feedback onset as 10*log10 (trial power/average baseline power). Power values were averaged across trials for every channel, condition and subject. In line with a previous study^11^, we investigated the effect of valence and probability on beta power and the distribution of these effects over the scalp for a subset of 9 representative electrode locations. To be specific, a 9 × 2 × 2 ANOVA was applied to beta power averaged over the 250–450 ms post-feedback interval with channel (F5, FZ, F6, C5, CZ, C6, P5, PZ, P6), valence (reward, error), and probability (high, low) as factors. Based on visual inspection, beta power was averaged within the 20–30 Hz range.

Source localization was performed with standardized low resolution electromagnetic tomography (sLORETA)^35^. For each subject, channel, and trial, a 2-second data segment spanning 1 s before feedback onset to 1 s after feedback onset was analyzed for time-varying cross-spectra in sLORETA with a 72 sample-long Gaussian window for the beta frequency range (20–30 Hz). Note that source localization cannot be conducted directly on power values, which are related to the square of the voltage values. Therefore, sLORETA brain maps were determined by recalculating the time-varying cross-spectral power values in the mid-frequency, 25 Hz, for each subject and condition. Statistically significant differences in beta power values were identified for each contrast by conducting paired t-tests for each voxel; the voxels with the largest t-values are reported. Randomization via statistical non-parametric mapping (SnPM) was applied in sLORETA to correct for multiple comparisons.

All error terms reported for the behavioral data constitute standard deviations.

## Additional Information

**How to cite this article**: Hosseini, A. H. and Holroyd, C. B. Reward feedback stimuli elicit high-beta EEG oscillations in human dorsolateral prefrontal cortex. *Sci. Rep.* **5**, 13021; doi: 10.1038/srep13021 (2015).
