# Erratum: Reward feedback stimuli elicit high-beta EEG oscillations in human dorsolateral prefrontal cortex

**DOI:** 10.1038/srep14400

**Published:** 2015-10-23

**Authors:** Azadeh HajiHosseini, Clay B. Holroyd

Scientific Reports 5: Article number: 1302110.1038/srep13021; published online: 08172015; updated: 10232015

The original HTML version of this Article contained a typographical error in the spelling of the author Azadeh HajiHosseini which was incorrectly given as Azadeh Haji Hosseini. This has now been corrected in the HTML version of the Article.

In addition, the original version of this Article quoted an incorrect abbreviation for Azadeh HajiHosseini in the ‘How to cite this article’ section. This has now been corrected in both the PDF and HTML versions of the paper.

